# The Soil–Plant Coupling Principle (SPCP): A Conceptual Framework for Diagnosing Hidden Ecosystem Vulnerability

**DOI:** 10.3390/plants15172572

**Published:** 2026-08-24

**Authors:** Adriano Sofo, Carmine Crecchio, Rosangela Addesso, Mohammad Yaghoubi Khanghahi

**Affiliations:** 1 Department of Agricultural, Forestry, Food and Environmental Sciences (DAFE), University of Basilicata, Via dell’Ateneo Lucano 10, 85100 Potenza, Italy; adriano.sofo@unibas.it (A.S.); rosangela.addesso@unibas.it (R.A.); 2 Department of Soil, Plant and Food Sciences, University of Bari Aldo Moro, Via Amendola 165/A, 70126 Bari, Italy; carmine.crecchio@uniba.it

**Keywords:** biological coupling, early-warning signals, ecosystem vulnerability, interaction-based diagnostics, soil microbiome, soil–plant interface

## Abstract

We introduce the Soil–Plant Coupling Principle (SPCP), a conceptual framework proposing that ecosystem stability emerges from the integrity of biological interactions rather than the condition of individual ecosystem components. Although current ecological assessments primarily rely on soil properties, plant performance, and microbial diversity, growing evidence indicates that ecosystems can remain apparently functional while progressively losing the coordination among processes that sustain resilience. This gap limits the ability of conventional diagnostics to capture interactional changes underlying early ecosystem vulnerability. Unlike ecosystem multifunctionality and resilience, which primarily describe the provision of multiple functions and the capacity to resist or recover from disturbance, respectively, SPCP focuses on the coordination among the processes that sustain these functions and resilience. SPCP reframes ecosystem degradation as progressive decoupling among carbon allocation, nutrient cycling, hydrological processes, and microbial interactions at the soil–plant interface. By integrating recent advances in soil ecology, plant physiology, microbiome science, ecological networks, and biogeochemistry, the framework provides a unified conceptual basis for understanding how interactional connectivity regulates ecosystem resilience. The objectives of this Perspective are to: (i) establish the theoretical basis for viewing ecosystem stability through soil–plant coupling; (ii) define the core dimensions of coupling integrity; and (iii) outline how coupling integrity could be operationalized using structural, functional, and interaction-based indicators. We further discuss the potential of coupling integrity as an early-warning property and identify key limitations and research needs for testing and validating the framework. By proposing an integrative and potentially testable framework, the manuscript provides a new lens for understanding ecosystem vulnerability and resilience across scales.

## 1. Introduction

Accelerating soil degradation, biodiversity loss, and climate instability increasingly reveal the limits of prevailing approaches for assessing ecosystem health. These pressures operate as interconnected drivers of systemic fragility, undermining the capacity of terrestrial ecosystems to sustain key functions such as carbon storage, nutrient retention, and hydrological regulation [1,2]. Despite major advances in ecosystem science, current frameworks remain largely compartmentalized, relying on isolated indicators such as soil nutrient status, plant productivity, or microbial diversity. However, this component-based perspective assumes that ecosystem stability can be inferred from the condition of individual subsystems, an assumption that is increasingly challenged.

A growing body of evidence indicates that ecological stability emerges from the integration of soil, plant, and microbial processes rather than from their independent performance. Soil–plant-microbiome interactions regulate key ecosystem functions through tightly coupled feedback mechanisms, where plant-derived carbon inputs structure microbial metabolism, and microbial transformations control nutrient availability and plant performance [3,4]. Recent studies further demonstrate that resilience depends on coordinated cross-compartment dynamics and interaction networks rather than on microbial diversity or biomass size alone [5,6]. Emerging work also highlights that microbial network stability, plant diversity, and soil physicochemical conditions co-evolve during ecosystem recovery, reinforcing the role of interaction dynamics in maintaining ecosystem function [7,8].

At the same time, current approaches often fail to detect early-stage ecosystem vulnerability because they overlook the coordination of biological interactions across the soil–plant-microbe interface. Ecosystems may maintain apparently stable levels of nutrients, productivity, or biodiversity while undergoing progressive degradation of the interactions that coordinate carbon allocation, nutrient transformation, hydrological exchange, and microbial activity. Soil systems, for example, can retain measurable nutrient stocks while losing aggregation and pore connectivity, constraining coordinated exchange processes [9]. Similarly, ecosystem multifunctionality studies show that the delivery of ecosystem services depends on coordinated interactions among processes, yet these dynamics remain poorly captured by existing metrics [10]. Advances in microbiome-informed restoration further emphasize that reinforcing biological interactions is central to ecosystem recovery, suggesting that coupling processes are critical but remain under-theorized [11,12].

These findings point to a fundamental gap in current ecological theory. Although ecosystem stability is increasingly recognized as an emergent property of interacting biological processes, existing frameworks provide no representation of the interactional architecture that coordinates these processes across the soil–plant interface. We argue that this regulatory architecture is maintained through dynamic coupling among carbon allocation, nutrient transformation, hydrological processes, and microbial activity, operating across spatial and temporal scales [13].

Here, we propose the Soil–Plant Coupling Principle (SPCP) as a unifying framework that reframes ecosystem stability as an emergent property of biological coupling at the soil–plant interface. Specifically, this perspective aims to establish the theoretical basis of SPCP, define the structural, functional, and feedback dimensions of coupling integrity, and outline approaches for its operationalization and measurement. We also examine its potential for improving the early detection of ecosystem vulnerability and identify the main limitations and empirical requirements for testing the framework.

From this framework, we interpret ecosystem vulnerability as progressive decoupling, an erosion of interactional coherence that precedes measurable declines in conventional indicators. We synthesize recent advances across soil ecology, plant physiology, and microbiome science to formalize coupling as a central organizing principle of ecosystem stability, and we outline a pathway for operationalizing coupling integrity through interaction-based diagnostics. By shifting the analytical focus from components to interactions, this Perspective provides a foundation for detecting early-warning signals of ecosystem degradation and for informing regenerative land management under accelerating global change.

SPCP builds on, but differs from, several established frameworks. Plant–soil feedback theory emphasizes reciprocal effects between plants and soil biota [14], while ecosystem multifunctionality focuses on the simultaneous provision of multiple ecosystem functions [6]. Ecological network theory, in turn, provides tools for describing the structure and stability of interactions among system components [15]. SPCP brings these perspectives together by focusing specifically on the coordination of soil, plant, and microbial processes at the soil–plant interface and by treating the integrity of these connections as a potential system-level property.

## 2. From Component-Based Assessment to Interaction-Driven Resilience

Current approaches to ecosystem assessment remain largely rooted in compartmental indicators, despite growing recognition that ecosystem responses to global change emerge from tightly coupled biological processes. Soil systems are commonly evaluated using nutrient stocks and physicochemical properties, plant systems through productivity and functional traits, and microbial communities through diversity and composition metrics [16,17]. Although these indicators provide valuable information about individual ecosystem components, they often fail to capture system-level dynamics governed by nonlinear feedback and cross-scale interactions [18,19,20].

Evidence increasingly suggests that ecosystems can maintain apparently stable component-level properties while progressively losing resilience [21]. Biodiversity loss, environmental change, and land-use intensification may reorganize ecological interactions without producing immediate declines in biomass or productivity, thereby masking early stages of ecosystem vulnerability [22,23,24]. This discrepancy highlights a critical limitation: component-based diagnostics are often insensitive to changes in interaction structure and the underlying “ecological wiring” that sustains system stability [25].

This interactional dimension is particularly evident in soil–plant–microbiome systems. Microbial communities form complex ecological networks whose connectivity, modularity, and redundancy influence ecosystem robustness and resistance to disturbance [26,27]. Likewise, coordinated feedback linking plants, soils, and microbial communities regulates ecosystem processes across aboveground and belowground compartments [4,14]. Disruption of these feedbacks, whether through land-use change, climate stress, or management practices, can destabilize ecosystem functioning even in the absence of immediate declines in diversity or productivity [28]. This decoupling of above- and belowground systems often serves as a precursor to rapid ecosystem state shifts [29,30]. Importantly, these findings support the central premise of SPCP that ecosystem stability is strongly influenced by interactional network organization, a dimension that current assessment frameworks do not formally capture. This raises an important question about whether changes in these interactions may become apparent before conventional measures of ecosystem function decline, which is considered in the following section.

## 3. Hidden Vulnerability and the Problem of Late Detection

A central implication of interaction-driven dynamics is that ecosystem degradation may proceed through a phase of hidden vulnerability, in which interactional coherence may erode before conventional indicators respond [31]. This phenomenon is increasingly supported by evidence from early-warning theory and empirical studies, showing that shifts in system dynamics, such as changes in autocorrelation, variance, and network structure, can precede observable changes in state variables [32,33]. Moreover, recent work highlights that such transitions may be associated with progressive decoupling of system interactions, reflecting underlying loss of coordination before collapse becomes detectable [34,35]. For instance, soil structural degradation, through the loss of aggregation and pore connectivity, can decouple water, gas, and nutrient fluxes while leaving bulk nutrient pools apparently unchanged, thereby obscuring early functional decline, as soil pore architecture directly regulates fluid transport, microbial activity, and biogeochemical exchange processes [17,36,37]. Similarly, climate-induced stress can disrupt plant-microbe feedbacks and carbon allocation, reducing system resilience before productivity decline becomes detectable [27].

From a systems perspective, these patterns align with early-warning theory, which suggests that critical transitions are often preceded by subtle changes in system dynamics, including increased variance, autocorrelation, and slower recovery rates, before any abrupt changes become apparent in state variables [38,39]. Despite this understanding, most ecological monitoring frameworks continue to emphasize state variables, with considerably less attention given to interaction dynamics. This imbalance limits their ability to detect early-warning signals and anticipate impending regime shifts [35,40]. These observations support the possibility that progressive decoupling may contribute to ecosystem vulnerability and may provide early-warning information, while highlighting the need for a unifying framework to integrate these processes into ecosystem assessment.

## 4. Theoretical and Empirical Foundations for Interaction-Based Resilience

Despite growing recognition of the importance of interactions in regulating ecosystem functioning, a clear link between interaction dynamics and ecosystem resilience is still missing from many assessment frameworks. Ecosystem multifunctionality metrics and biodiversity indicators capture important aspects of system performance, but do not quantify the coherence, coordination, or stability of interactions across components [6,41]. This limitation is particularly relevant when ecosystem functions remain apparently stable despite changes in the interactions that support them. More recent work further emphasizes that multifunctionality and ecosystem services emerge from complex interaction networks among soil, plants, and microbial communities, yet these relationships are typically inferred indirectly [42,43]. As a result, current frameworks remain fundamentally limited in diagnosing interactional coordination, leaving a critical gap in detecting early-stage ecosystem vulnerability.

A growing body of ecological theory and empirical evidence suggests that resilience depends not only on the condition of individual components, but also on the organization and stability of the relationships among them. In microbial communities, for example, network connectivity, modularity, and functional redundancy can influence resistance to environmental disturbance and the persistence of ecosystem functions [26,27]. At the same time, interactions between plants, soil organisms, and their physical environment regulate the transfer of carbon, nutrients, and water across ecosystem compartments [4,14]. These interactions provide a mechanistic basis for understanding why changes in one component may propagate through the system even when conventional indicators remain relatively stable.

Empirical studies also show that interaction-based processes can be examined using complementary approaches. Stable isotope tracing and metabolomics can reveal carbon and nutrient flow among interacting biological compartments [44,45], whereas network-based analyses can describe interaction structure and identify changes in connectivity or interaction strength [46]. Soil structural measurements provide an additional dimension by describing the physical pathways through which water, gases, nutrients, and organisms move. However, despite these methodological advances, most studies rely on these approaches independently, with each capturing only one aspect of ecosystem interactions. Stable isotope tracing, network analysis, and biogeochemical measurements therefore provide valuable but partial perspectives that are rarely integrated into a unified diagnostic framework. Consequently, current applications characterize specific interaction processes, such as carbon fluxes, nutrient transformations, or network topology, but provide limited insight into how these processes are coordinated across the system as a whole.

The resulting gap is therefore not a lack of methods for studying individual interactions, but the absence of a framework that connects these observations into a common measure of ecosystem-level coordination. Such integration is needed to move from describing individual processes toward evaluating whether the interactions among soil, plants, and microorganisms remain coherent under environmental change.

## 5. From Hidden Vulnerability to a Coupling-Based Framework

Taken together, current evidence converges on three key insights:

(i) Ecosystem stability cannot be reliably inferred from component-level indicators alone
(ii) Interaction networks and feedback coherence are central to resilience
(iii) Ecosystem degradation may originate as progressive decoupling that remains undetected by existing metrics

These observations help connect the problem of late detection described in Section 3 with the need for a more integrative diagnostic framework. If ecosystem vulnerability develops partly through a progressive loss of coordination, then measuring the condition of individual components is not sufficient to identify this process. What is needed is an approach that considers how soil, plants, and microbial communities remain connected through the exchange of carbon, nutrients, water, and biological signals.

This perspective does not imply that conventional ecosystem indicators should be replaced. Rather, interaction-based measurements can complement them by providing information on the organization and coordination of processes that may change before major shifts in ecosystem state become apparent. The key challenge is therefore to translate the concept of interactional coherence into measurable properties that can be compared across environmental conditions and disturbance regimes.

This conceptual need provides the basis for the Soil–Plant Coupling Principle (SPCP). In the following section, we develop SPCP as a framework for describing ecosystem stability in terms of the integrity of biological coupling at the soil–plant interface. The framework links structural properties, ecosystem processes, and reciprocal feedbacks, providing a conceptual basis for developing interaction-based indicators of ecosystem vulnerability and early-warning signals.

## 6. The Soil–Plant Coupling Principle (SPCP)

### 6.1. Core Principles of SPCP

The Soil–Plant Coupling Principle (SPCP) holds that terrestrial ecosystem stability is governed by the integrity of biological coupling at the soil–plant interface, where carbon allocation, nutrient transformation, hydrological processes, and microbial interactions are dynamically coordinated. Under SPCP, ecosystem functioning is not determined by the independent performance of soil, plant, or microbial subsystems, but by the coherence of feedbacks that link them across spatial and temporal scales. In this view, coupling integrity refers to the capacity of these components and processes to remain functionally connected and coordinated under changing environmental conditions. A progressive loss of this coordination may weaken system resilience before measurable changes become evident in conventional indicators such as soil properties, plant productivity, or microbial diversity. SPCP thus shifts the focus from the condition of individual components toward the organization and coherence of the interactions that connect them.

The novelty of SPCP therefore lies not in introducing new individual mechanisms, but in integrating these established processes into a common framework in which coupling integrity is treated as a measurable system-level property.

### 6.2. Theoretical Foundations and Supporting Evidence

This perspective aligns with recent advances in microbial ecology and biogeochemistry, which demonstrate that plant-derived carbon inputs regulate microbial metabolism and network structure, while microbial transformations control nutrient availability and feedback to plant performance [43]. Similarly, emerging work shows that the structure and stability of microbial interaction networks influence ecosystem-level processes such as carbon cycling and resilience to disturbance [23].

**Figure 1:**
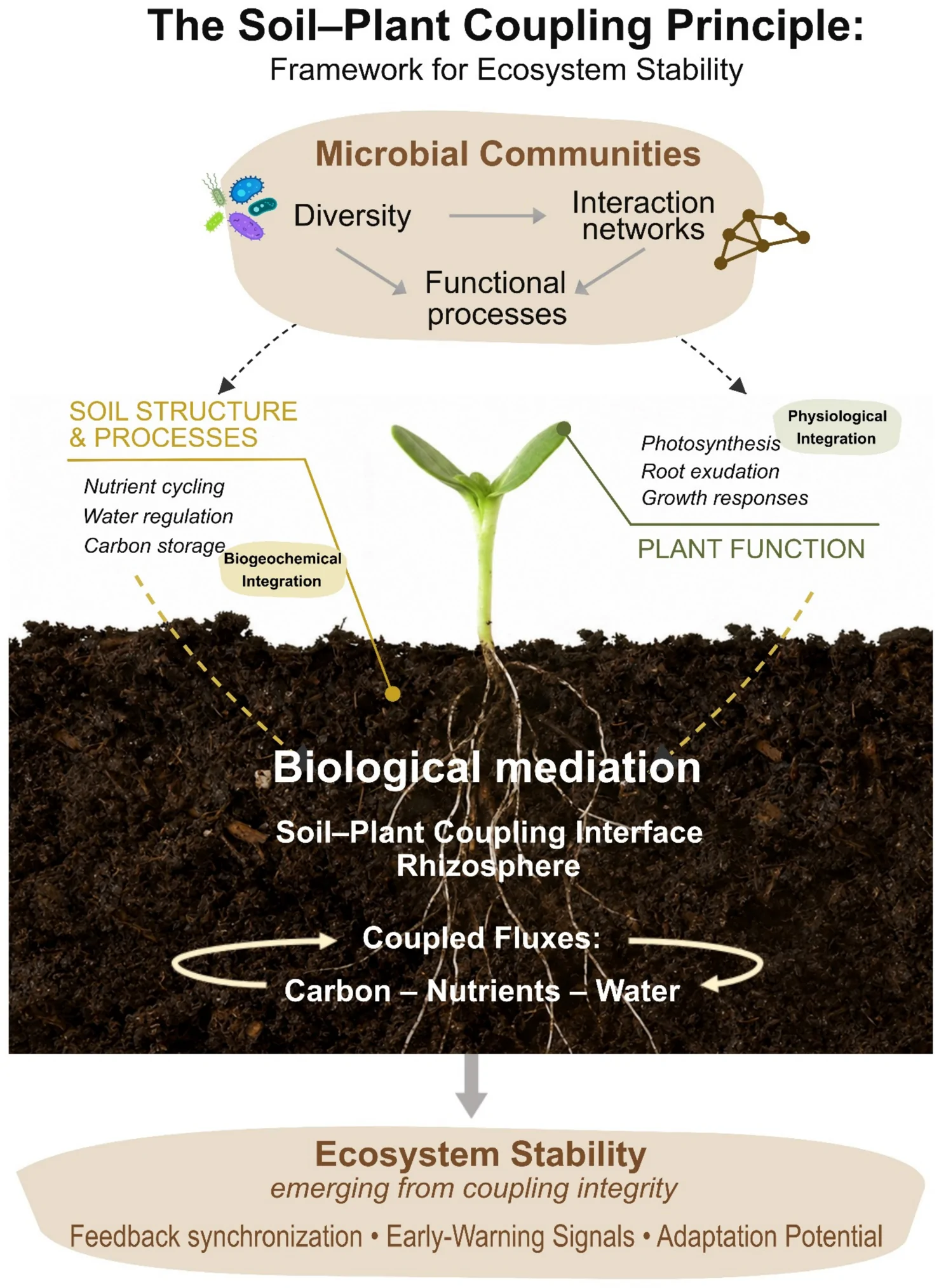
Conceptual architecture of the Soil–Plant Coupling Principle (SPCP). Biological coupling at the soil–plant interface integrates soil structure and biogeochemical processes, plant physiological functions, and microbial communities through coordinated carbon, nutrient, and water fluxes. Within this framework, ecosystem resilience emerges from the integrity of these coupled interactions, while feedback synchronization provides the basis for adaptive responses and early-warning signals of ecosystem vulnerability.

Our previous studies provided an important empirical basis for developing the SPCP concept, particularly by revealing coordinated changes across soil properties, microbial communities, and plant responses under different environmental and management conditions. However, these studies represent only one part of the broader evidence supporting the interaction-centered perspective. These studies showed that changes in soil functioning are consistently accompanied by coordinated shifts in microbial community composition, functional gene abundance, soil physicochemical properties, and plant performance [31,47,48,49,50,51]. For example, long-term management practices simultaneously altered soil chemistry, microbial community organization, functional gene abundance, and biological activity, revealing coordinated system-wide responses. Likewise, studies across agricultural systems demonstrated that sustainable management reshapes soil organic matter dynamics, aggregate organization, microbial communities, and key soil functions through interconnected biological and physicochemical processes [52,53]. More recently, evidence has highlighted the role of microbial exopolysaccharides in linking microbial activity with soil aggregation, water retention, nutrient cycling, and plant performance, further supporting the importance of biological connectivity across the soil–plant interface [54]. Collectively, these findings reinforce the central premise of SPCP that ecosystem resilience is better understood through the integrity of biological interactions than through isolated measurements of individual ecosystem components. The conceptual architecture of SPCP is summarized in Figure 1.

Empirical evidence reinforces this relational interpretation. Soil management systems differentially regulate microbial genetic diversity and C/N dynamics [55,56]. These shifts alter not merely microbial abundance but the structure of belowground interaction networks. Management-driven changes modulate metabolomic profiles of xylem sap and plant physiological responses [56,57,58], demonstrating that plant function reflects restructuring of the soil–plant interface rather than simple nutrient concentration differences. Earthworm-mediated modifications of soil physical properties reshape root morphology and plant development [50], highlighting the importance of structural–biological integration. Carbon isotope discrimination under drought conditions reveals that water-use efficiency and carbon allocation depend on coordinated soil–plant regulation [59]. These findings converge on a consistent pattern: resilience depends on coupling integrity.

It should be noted that the processes underlying soil–plant coupling are broadly relevant across terrestrial ecosystems; however, an important next step is to test whether coupling integrity can be consistently quantified across different environmental contexts, ecosystem types, and disturbance regimes.

### 6.3. The Three Dimensions of Coupling

Building on these developments, SPCP organizes biological coupling into three complementary dimensions: structural, functional, and feedback coupling. These dimensions provide a conceptual basis for evaluating the coordination of soil, plant, and microbial processes across the soil–plant interface. This framework also offers a foundation for developing interaction-based indicators of ecosystem vulnerability.

(i) Structural coupling describes the physical and biological architecture that enables interactions, including soil aggregation, pore connectivity, and microbial network topology. These structural properties determine how resources, organisms, and signals move through the system.
(ii) At the process level, functional coupling reflects the coordination of key biogeochemical processes, including carbon allocation, nutrient transformation, and water flux regulation. High functional coupling reflects synchronized ecosystem processes that collectively sustain system functioning under changing environmental conditions.
(iii) A third dimension, feedback coupling, describes the strength and stability of reciprocal interactions linking plants and soil communities, particularly plant–microbe feedbacks and carbon-mediated regulatory loops that shape ecosystem responses to disturbance.

### 6.4. Operationalization and Measurement Approaches

A central implication of SPCP is that coupling integrity can be treated as a measurable system property. Within this framework, the emphasis extends beyond state variables (e.g., nutrient pools and biomass) to the coordination among processes that regulate ecosystem dynamics. Recent methodological advances have made this perspective increasingly feasible. Network-based approaches can quantify interaction topology, including connectivity, modularity, and robustness, all of which are closely associated with ecosystem functioning [27,60]. Stable isotope tracing enables direct measurement of carbon flow pathways between plants and microbial communities, providing insights into feedback strength and resource allocation [44]. In parallel, coupled biogeochemical analyses can assess the synchronization of carbon and nutrient cycles, a key feature for maintaining ecosystem function under environmental stress [43]. Together, these approaches provide the basis for translating interaction dynamics into measurable indicators of coupling integrity.

**Figure 2:**
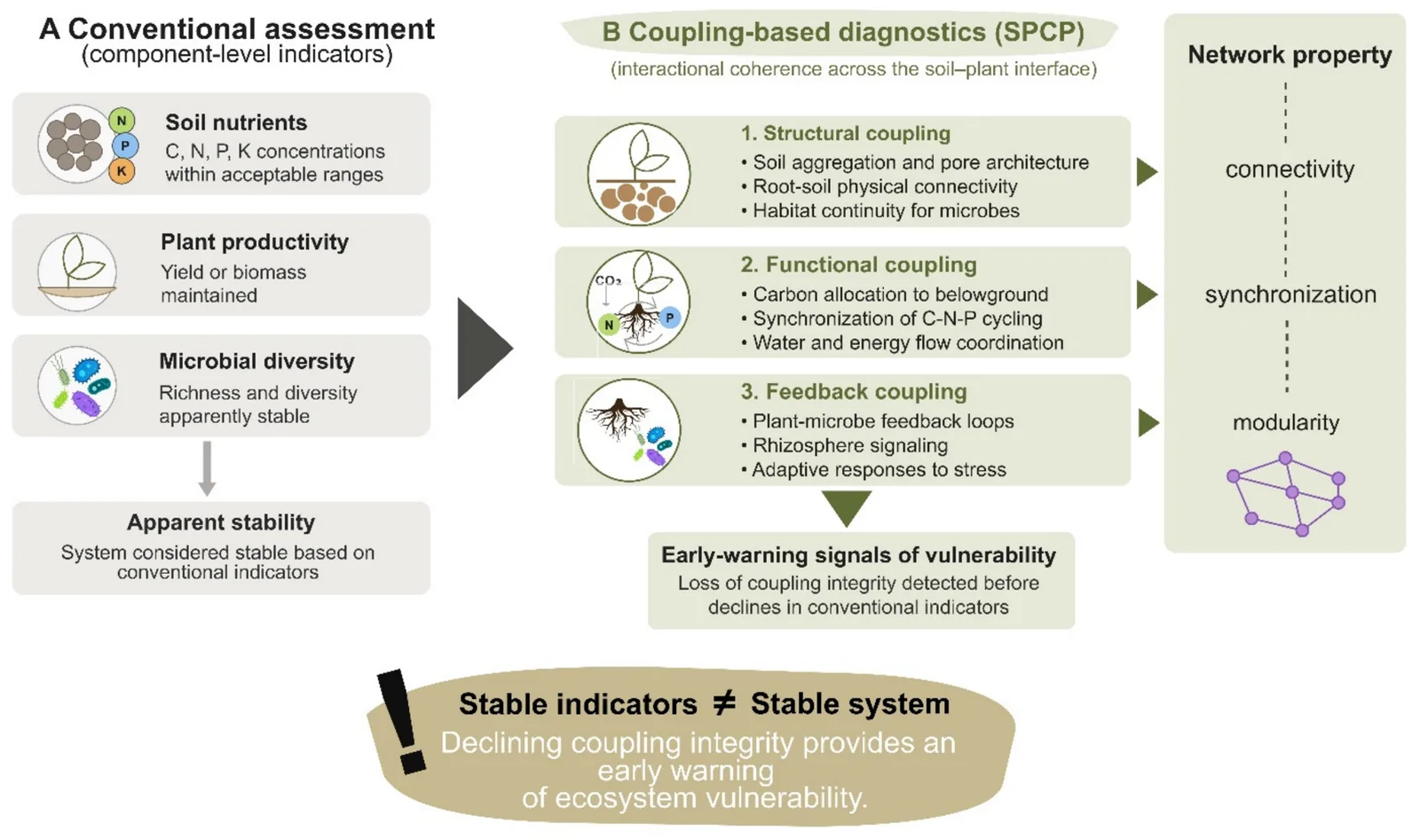
From component-based assessment to coupling-based ecosystem diagnostics. Conventional ecosystem assessment (**left**) infers system stability from component-level indicators such as soil nutrient status, plant productivity, and microbial diversity. In contrast, the SPCP framework (**right**) evaluates ecosystem condition through interactional coherence across structural, functional, and feedback coupling. Progressive loss of coupling integrity provides early-warning signals of ecosystem vulnerability before detectable changes occur in conventional state variables.

These dimensions can be quantified by integrating complementary measurements from soil physics, microbial ecology, ecological network analysis, stable isotope tracing, and biogeochemistry. Together, they provide the basis for coupling-based indicators that capture interactional organization and may provide earlier evidence of ecosystem vulnerability than conventional state variables. This possibility is supported by growing evidence that disruption of microbial networks and feedback processes can precede detectable losses of ecosystem function [23,27]. Figure 2 summarizes how the conceptual framework can be translated into coupling-based diagnostics by substituting conventional component-level indicators with interaction-based assessment across structural, functional, and feedback dimensions.

Coupling integrity could be assessed by combining complementary indicators within the three dimensions of SPCP. Structural coupling could be evaluated from soil aggregation, pore connectivity, root distribution, and microbial network properties such as connectivity and modularity. Functional coupling could be assessed through coordinated carbon, nutrient, and water fluxes using measurements such as stable isotope tracing, nutrient transformation rates, and plant–soil exchange processes. Feedback coupling could be evaluated from the strength and stability of reciprocal plant-microbe and soil–plant relationships, including their responses to environmental perturbations. These measurements could then be integrated into a multidimensional coupling profile, allowing changes in the coordination among components to be compared across sites, management regimes, or disturbance gradients.

Importantly, SPCP does not require a single universal coupling index. Coupling integrity may instead be represented as a multidimensional profile, with the relative contribution of structural, functional, and feedback dimensions evaluated according to ecosystem context and the disturbance being considered. A composite index could be developed where appropriate, but its formulation and weighting would require empirical validation across ecosystems.

### 6.5. Limitations and Boundary Conditions

SPCP is not intended to replace component-level indicators in all situations. Conventional indicators may remain sufficient when ecosystem degradation is already severe, when ecosystem structure is highly simplified, or when the assessment objective concerns a specific component or function. The added value of coupling-based diagnostics is greatest in systems where individual components remain apparently functional, but their interactions may be changing in ways that are not captured by component-level measurements. Thus, SPCP should be viewed as complementary to, rather than a replacement for, established ecosystem indicators.

A further challenge is distinguishing changes in coupling from changes driven by shared environmental factors or other confounding variables. Temperature, soil moisture, nutrient availability, management intensity, and disturbance history, for example, may simultaneously affect several components of the soil–plant system and generate apparent changes in coupling. Robust application of SPCP will therefore require appropriate experimental designs, temporal replication, and multivariate or causal modelling approaches that account for major environmental drivers when interpreting coupling metrics.

An additional conceptual challenge concerns the direction of causality between decoupling and vulnerability. SPCP proposes that progressive loss of coupling may precede detectable declines in ecosystem function, but decoupling may also emerge as a consequence of environmental stress or declining ecosystem performance. These processes may therefore form reciprocal feedbacks. Distinguishing whether decoupling is a cause, consequence, or reinforcing mechanism of vulnerability will require longitudinal observations and experimental perturbations that can establish temporal precedence and test causal relationships.

The temporal relationship between coupling loss and functional decline is also unlikely to follow a single universal pattern. Coupling disruption may precede detectable functional decline under some stressors, whereas in other cases both may develop concurrently or functional changes may emerge before measurable changes in coupling. The lag between changes in coupling and conventional ecosystem indicators is therefore expected to depend on ecosystem characteristics, disturbance type, stress intensity, and the response variable being monitored. At present, there is insufficient evidence to propose a general lag time for SPCP. Establishing such temporal relationships will require repeated measurements before, during, and after disturbance across different ecosystem types and stress regimes.

## 7. Strengths, Challenges, and Pathways for Validation

The perspective of SPCP is consistent with growing evidence that soil microbiomes and plant-associated microbial communities regulate ecosystem multifunctionality through interconnected biological processes, explaining substantial variation in productivity, nutrient cycling, and enzymatic activity across environmental gradients [10]. Similarly, recent studies show that microbial communities function as complex interaction networks in which mutualistic, competitive, and trophic relationships collectively shape ecosystem resilience [61]. These findings support the central premise of SPCP that the organization and integrity of interactions, beyond the abundance or diversity of individual components, are fundamental determinants of ecosystem persistence.

An important advantage of SPCP is its ability to integrate emerging methodological advances, including multi-omics, high-resolution soil imaging, stable isotope tracing, and ecological network analysis, within a single conceptual framework. By linking these complementary approaches, SPCP provides a foundation for moving beyond descriptive ecosystem assessments toward interaction-based diagnostics that explicitly quantify the mechanisms underlying resilience and vulnerability.

At the same time, several challenges must be addressed before coupling-based diagnostics can be widely implemented. Biological interactions are inherently context-dependent, varying with environmental conditions, dispersal processes, and stochastic community assembly, making it difficult to derive universally applicable measures of coupling [62]. Within SPCP, this context dependence can be understood as a modulation of coupling relationships along environmental gradients. Changes in factors such as soil moisture, temperature, nutrient availability, disturbance intensity, or management can alter the strength, direction, and stability of interactions among soil, plants, and microorganisms [63]. This perspective allows the specific magnitude and composition of coupling relationships to vary among ecosystems while maintaining a common focus on how their strength, stability, and organization respond to environmental change.

A pathway toward a more generalizable SPCP framework is to distinguish context-dependent metrics from more general properties of coupling. Network connectivity, robustness, interaction stability, synchronization among coupled processes, and the persistence of functional linkages may represent candidate properties that can be evaluated across systems, even when their absolute values differ among ecosystems. Rather than seeking universal numerical thresholds, cross-system comparisons could focus on the direction, persistence, and coordinated change in these properties relative to ecosystem-specific reference states. This approach would allow SPCP to retain common principles while remaining flexible to ecological context.

Methodological fragmentation also remains a major obstacle. Although isotope tracing, network analysis, and soil structural characterization are often applied separately, they provide complementary information about ecosystem interactions. They are rarely integrated into unified analytical frameworks capable of diagnosing system-level behavior [64]. Furthermore, land-use change and other anthropogenic pressures can reorganize microbial networks in nonlinear ways, complicating the interpretation and transferability of coupling metrics across ecosystems [47,65]. Bridging the remaining gap between research methodologies and operational ecosystem monitoring is therefore an important priority for future work.

Despite these challenges, SPCP provides a basis for several priorities for future research and generates testable hypotheses linking network organization to ecosystem resilience. Recent studies indicate that microbial interaction networks explain substantial variation in ecosystem multifunctionality and respond sensitively to environmental disturbance, suggesting that interaction-based metrics can serve as early indicators of ecosystem reorganization [10,11,61,62]. Additional integrative research further emphasizes the need to connect microbial interactions, nutrient cycling, and environmental drivers to improve predictions of ecosystem responses to global change [8]. These predictions can be evaluated through experimental manipulation of coupling processes, long-term monitoring of interaction networks, and the integration of multi-omics with biogeochemical measurements. Collectively, these approaches provide a practical pathway for validating coupling-based diagnostics and incorporating them into predictive frameworks for ecosystem assessment. Such comparative and longitudinal approaches could also help determine whether coupling loss consistently precedes functional decline, estimate the duration of any lag between these responses, and identify how these temporal relationships vary across ecosystems and disturbance regimes. Key priorities include testing the framework across contrasting ecosystems and disturbance regimes, establishing temporal benchmarks for coupling loss, and determining which coupling properties are most consistently associated with ecosystem resilience.

## 8. Conclusions and Outlook

Accelerating environmental change demands new ways of understanding ecosystem stability that extend beyond conventional component-based assessments. In this Perspective, we propose the Soil–Plant Coupling Principle (SPCP) as a conceptual framework that places biological interactions, rather than isolated ecosystem components, at the center of ecosystem regulation. By viewing stability as an emergent property of coordinated coupling among carbon allocation, nutrient cycling, hydrological processes, and microbial interactions, SPCP therefore offers a mechanistic perspective on how ecosystems may lose resilience while conventional indicators still appear stable. It highlights coupling integrity as a potentially important property for ecosystem assessment.

Beyond its conceptual contribution, SPCP provides a foundation for developing interaction-based diagnostics capable of identifying hidden ecosystem vulnerability. Advances in microbial ecology, ecological network analysis, stable isotope tracing, and soil imaging increasingly make it possible to quantify the integrity of biological coupling across multiple organizational levels. Integrating these complementary approaches represents an important step toward detecting ecosystem instability before irreversible functional change occurs.

Several priorities now emerge for testing and developing the framework. Standardized metrics of coupling integrity are needed to enable comparisons across ecosystems and environmental gradients. Experimental and long-term observational studies should evaluate whether progressive decoupling consistently precedes measurable declines in ecosystem functioning and determine the predictive value of coupling-based indicators under different disturbance regimes. Extending this framework to Earth system processes, including carbon sequestration, nutrient retention, and hydrological regulation, will further clarify how local interaction dynamics scale to regional and global ecosystem stability. Translating these concepts into practical monitoring tools will be equally important for supporting adaptive ecosystem management under accelerating global change.

More broadly, SPCP shifts attention from measuring ecosystem components in isolation to understanding the interactional architecture that organizes ecological processes across scales. If ecosystem resilience emerges from the coherence of these interactions, then identifying, quantifying, and monitoring coupling integrity becomes a central challenge for ecosystem science. We therefore encourage the ecological community to test this framework across ecosystems, stress regimes, and spatial and temporal scales, and to determine which aspects of coupling provide reliable information about resilience and early vulnerability. Such efforts can help move ecosystem assessment from predominantly component-based measurements toward more predictive and interaction-aware approaches to ecosystem management.

## Data Availability

No new datasets were generated or analyzed during the current study. Therefore, data sharing is not applicable to this article.
